# Zearalenone: A Mycotoxin With Different Toxic Effect in Domestic and Laboratory Animals’ Granulosa Cells

**DOI:** 10.3389/fgene.2018.00667

**Published:** 2018-12-18

**Authors:** Guo-Liang Zhang, Yu-Long Feng, Jun-Lin Song, Xiang-Shan Zhou

**Affiliations:** ^1^Qingdao Agricultural University, Qingdao, China; ^2^National Engineering Research Center for Gelatin-based Traditional Chinese Medicine, Dong-E-E-Jiao Co. Ltd., Liaocheng, China; ^3^State Key Laboratory of Bioreactor Engineering, East China University of Science and Technology, Shanghai, China

**Keywords:** zearalenone, toxicity, swine, *Equus asinus*, granulosa cells

## Abstract

Zearalenone (ZEA), one of the most prevalent estrogenic mycotoxins, is mainly produced by *Fusarium* fungi and has been proven to affect the reproductive capacity of animals. Exposure of farm animals to ZEA is a global public health concern because of its toxicity and wide distribution in animal feeds. *In vitro* and *in vivo* experiments indicate that ZEA possesses estrogenic activity in mice, swine, *Equus asinus* and cattle. The precise mechanism of the reproductive toxicity of ZEA has not been established yet. This article reviews evidence on the deleterious effects of ZEA on mammalian folliculogenesis from early to final oogenesis stages. Such effects include impaired granulosa cell (GC) development and follicle steroidogenesis, reduced oocyte nest breakdown, damaged meiotic progression, poor fetal oocyte survival, accelerated primordial follicle activation and enhanced follicle atresia. These phenomena may result in reproductive and non-reproductive problems in domestic animals. In addition, emerging data indicates that ZEA may cause mRNA expression changes in the GCs. In general, *E. asinus* is more sensitive than swine to ZEA exposure. Finally, results of *in vivo* animal studies and *in vitro* tests are reported and discussed.

## Introduction

**FIGURE 1:**
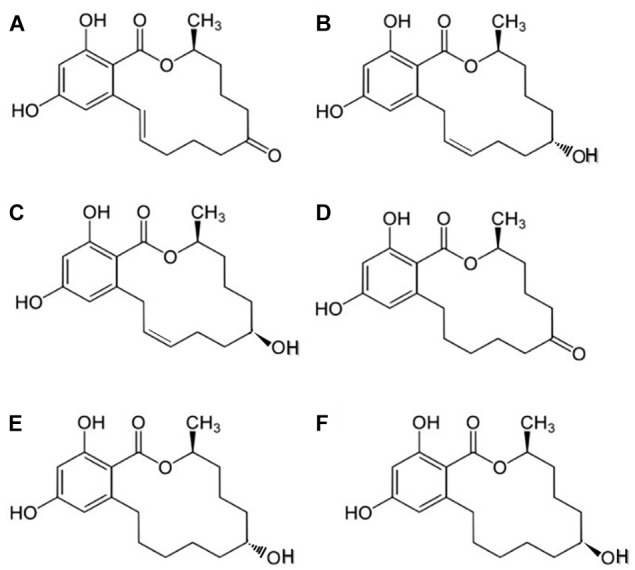
Chemical structures of ZEA and its derivatives: **(A)** zearalenone (ZEA), **(B)** α-zearalenol (α-ZEA), **(C)** β-zearalenol (β-ZEA), **(D)** zearalanone (ZAN), **(E)** α-zearalanol (α-ZAL), and **(F)** β-zearalanol (β-ZAL).

Zearalenone (ZEA) is a mycotoxin produced by the *Fusarium* species (*F. graminearum*, *F. cerealis*, etc.), which are distributed worldwide (Bennett and Klich, 2003). ZEA or 6-(10-hydroxy-6-oxo-trans-1-undeceny) β-resorcylic acid lactone has a molecular formula of C_18_H_22_O_5_ (Figure 1; Kuiper-Goodman et al., 1987). Toxigenic species, such as *Fusarium* fungi, including *F. graminearum (Gibberella zeae), F. culmorum, F. cerealis, F. equiseti, F. crookwellense*, and *F. semitectum*, are major pathogens of cereal plants and cause ear rot in maize and head blight in wheat and barley (Kuiper-Goodman et al., 1987). ZEA is typically detected in high levels in samples of natural animal feeds, because of their improper storage, even though, toxigenic *Fusarium* species infect cereals and lead to ZEA accumulation before the harvest time (D’Mello et al., 1999). Earlier investigations of Placinta et al. (1999) showed that *Fusarium* fungi spread from one country to another with increased grain trade worldwide (Placinta et al., 1999).

Researchers have investigated the metabolism of ZEA in mammals (Olsen et al., 1981) and reported that the keto group on C-8, is degraded into two metabolites (α- and β-ZEA). These metabolites can also be produced by *Fusarium*, but at lower concentrations compared with that generated from ZEA (CCFAC, 2000). The two major principal biotransformation pathways for ZEA in mammals are as follows: formation of α- or β-ZEA through hydroxylation, catalyzed *in vivo* by 3α- and 3β-hydroxy-steroid-dehydrogenases (HSDs); and conjugation of ZEA with glucuronic acid, catalyzed by uridine diphosphate glucuronyl transferases (Zinedine et al., 2007).

Zearalenone naturally occurs in agricultural crops, particularly in maize. This mycotoxin could contaminate products made of barley, wheat, oats, rice, and sorghum. Given its prevalence and heat stability (up to 160°C) (Kuiper-Goodman et al., 1987), ZEA cannot be completely eradicated in the feed chain. Although ZEA is non-steroidal, ZEA and its derivatives act similarly to 17β-estradiol (E_2_) by inhibiting the secretion and release of steroid hormones, thus disrupting endogenous estrogenic response during the preovulatory stage and depressing the maturation of ovarian follicles (Katzenellenbogen and Korach, 1997). The estrogenic activity of ZEA causes several reproductive disorders in domestic animals as well as hyperestrogenic syndromes in humans (Poor et al., 2015), which depend on the dose and time of exposure.

### Effect of ZEA on the Reproduction of Swine and Other Domestic Animals

China is the world’s largest pork producer and responsible for nearly 50% of the world’s total pork production (Zhou and Yang, 2010). However, the pork industry has been heavily affected by ZEA. Kuiper-Goodman et al. (1987) reported that pigs are more sensitive to the reproductive effects of dietary ZEA than other domestic animals (Kuiper-Goodman et al., 1987) and there is currently no effective antidote for these toxins (Cortinovis et al., 2013). The toxic effects of ZEA on weaned gilts are associated with vulvar hypertrophy and ovarian atrophy but not with mammary and uterine enlargement (Vanyi et al., 1994). ZEA causes sterility in sows by inciting ovarian disorders (Obremski et al., 2003). Oocytes die in the follicles and ovulation does not occur, despite signs presented during the estrus cycle (Zwierzchowski et al., 2005). ZEA, similar to 17β-E_2_, inhibits the secretion of steroid hormones, disrupts estrogenic responses during the preovulatory stage, and suppresses the maturation of mammalian ovarian follicles (Katzenellenbogen and Korach, 1997). Changes in the estrous cycle, caused by ZEA, depend on its dose and administration time (Katzenellenbogen and Korach, 1997). In young swine, orally administered ZEA is rapidly absorbed and metabolized. The uptake of ZEA is 80–85% in swine given an oral dose of 10 mg/kg body weight (Biehl et al., 1993). Ueno et al. (1983) showed that α-ZEA is a major metabolite, at pH 7.4 and at pH 4.5, in the cultured oocytes of pigs (Ueno et al., 1983). Malekinejad et al. (2006) reported differences in ZEA biotransformation among different species; ZEA is predominantly catalyzed into α-ZEA in swine, while β-ZEA is the main hepatic metabolite in cattle (Malekinejad et al., 2006). The mechanism of α-ZEA in swine can be explained by its effect on cells in target tissues, while competing with the estrogen receptor (ER; Alm et al., 2002). ZEA including α-ZEA and β-ZEA was detected in the natural follicular fluid in porcine ovaries, through liquid chromatography–tandem mass spectrometry (Lai et al., 2015). The concentrations of ZEA and α-ZEA in porcine follicular fluid were found to be 38.9 and 17.6 pg/mL, respectively (Sambuu et al., 2011).

Flowers et al. (1987) reported long estrus intervals in cycling gilts that were given 20 mg of ZEA on days 6 to 10 or days 11 to 15 of the estrous cycle (Flowers et al., 1987). The estrous cycle was extended in gilts after receiving 10 mg/kg ZEA from day 5 to 20 of the cycle (Edwards et al., 1987; Minervini and Dell′Aquila, 2008). In another study, gilts were fed a diet containing 3.61 or 4.33 mg/kg ZEA from puberty to mating stages (Etienne and Jemmali, 1982). About 45% of these gilts developed pseudopregnancies induced by ZEA. Moreover, ZEA suppressed pig oocyte progression through meiosis by inducing the malformation of meiotic spindles (Malekinejad et al., 2007). Alm et al. (2006) reported that feeding gilts with wheat, naturally contaminated with ZEA, might interfere with the initial chromatin status and maturation competence of oocytes *in vitro* (Alm et al., 2006).

Zhu et al. (2012) reported that high concentrations (30–120 mM) of ZEA induced the apoptosis and impaired the proliferation of porcine granulosa cells (GCs) in a dose dependent manner (Zhu et al., 2012). In recent years, Qin et al. (2015) reported that ZEA caused the disorder of the mitochondrial transmembrane and increased the reactive oxygen levels in porcine GCs (Qin et al., 2015). Exposure to high levels of ZEA induces apoptosis or necrosis through the caspase-3/caspase-9 dependent mitochondrial pathway in porcine GCs. Thus, ZEA and its metabolites may induce atresia in porcine follicles.

*Equus asinus*, a kind of domestic animal that serves as a pet or draft animal, is important in mule and milk production worldwide (Canisso et al., 2010; Canisso et al., 2011). China has the richest *E. asinus* genetic resources and has a long history of raising *E. asinus* for agriculture and transportation (Cong et al., 2018; Zhu and Weng, 2018). Furthermore, *E. asinus* is the major source of the Chinese traditional tonic ejiao (Kumeta et al., 2014), which has considerable high economic value in China. The economic value of *E. asinus* prompts the development of assisted reproductive techniques. *E. asinus* is reported to be less sensitive to ZEA exposure than pigs. ZEA and its metabolites were detected in the bile of *E. asinus* (Diekman and Green, 1992). Moreover, ZEA is associated with infertility, hyperestrogenism, and reduced milk production in cows (Minervini and Dell′Aquila, 2008). Conception rates decreased from 87 to 62% when dairy heifers were fed 250 mg of 99% purified ZEA daily (Weaver et al., 1986). *In vitro* studies reported a significant decrease in the maturation rates of oocytes exposed to 3.1 mM ZEA. About 50% (62/124) of the matured oocytes were arrested in metaphase I after ZEA treatment (Takagi et al., 2008). ZEA exposure may also inhibit the production of estradiol in bovine GCs (Pizzo et al., 2015).

In cycling mares, ZEA and its metabolites have only been reported in a few cases. Juhasz et al. (2001) reported the effect of a 10-day low-dose ZEA exposure on the reproductive parameters (Juhasz et al., 2001). The treatment did not affect the duration of follicular phases and inter ovulatory intervals in mares that were fed 7 mg of ZEA daily, for 10 days after ovulation. Additionally, no significant differences were reported with regard to the influence of ZEA on follicular activity (size of ovulatory follicles, growth rate, maximum number and initial increase in the number of large follicles) (Juhasz et al., 2001). In a subsequent study, feeding oats naturally contaminated with ZEA (2 mg/kg) did not exert considerable effects on the length of the cycle and the release of reproductive hormones in mares (Cortinovis et al., 2013). However, these doses of ZEA are ineffective in sensitive species, such as swine (Obremski et al., 2003). *E. asinus*, that were fed naturally ZEA-contaminated oats, had a high incidence of follicular hematomas, which did not occur during control cycles (Cortinovis et al., 2013). ZEA and its derivatives induced the apoptosis of GCs collected from the ovaries of cycling mares *in vitro* (Minervini et al., 2006) and from cycling pigs (Zhang et al., 2017b). Considering the contemporaneous presence of both processes, authors suggested that ZEA could induce follicular atresia in domestic animals. In addition, these effects could be due to the direct interaction with ERs and the interaction with the enzymes of 3α/β HSD in the GCs and the ovaries, which is associated with the metabolism of endogenous steroid hormones (Minervini et al., 2006). Therefore, ZEA and its derivatives could induce ovarian follicular atresia in pigs and mares.

Limited information is available regarding the effects of ZEA on mammalian ovarian folliculogenesis in domestic animals (Cortinovis et al., 2013), particularly the effect on follicle formation and development (Table 1). Surveys with regard to the influence of ZEA exposure on the reproductive system of farm animals have primarily focused on fertility symptoms; however, the modes of action of ZEA have not been investigated yet. The reported mechanisms include the activation of the ERs and the androgen receptors (Tabacova et al., 1999).

**Table 1:** Some reproductive and disorders effects induced by ZEA in animals.

Animal type	Dosage and ZEA source	Duration	Effects	Reference
Sows	Natural contamination or addition of pure ZEA	–	Vulvovaginitis, anestrus, decreased luteinising hormone and progesterone secretion	Etienne and Dourmad, 1994
Sows, gilts and piglets	Fusarium-contaminated feed	–	Reduced conception rates, enlargement of ovaries and uterus, swelling of vulva in piglets	Vanyi et al., 1994
Horses	Natural contamination of 2.6 mg/kg	–	Outbreak of ZEA mycotoxicosis	Minervini et al., 2006
Cows	250 mg of 99% purified ZEA	1 days	Infertility and reduced milk production	Weaver et al., 1986
Rats	1.5, 3, and 5 mg/kg ZEA	–	Changes in some blood and biochemical parameters indicating liver toxicity	Maaroufi et al., 1996
Mice	5–30 μm of pure ZEA/animal	1–10 days	Mimic oestrogen actions, delayed vaginal opening, persistent oestrus and sterility	Ito and Ohtsubo, 1994
Gilts	200 μg ZEA/kg b.w.	8 days	Disturbances in the process of development and maturation of some of the ovarian follicles	Zwierzchowski et al., 2005

### Effects of ZEA on Primordial Follicle Assembly

After a brief overview of ZEA toxicity, this article describes the detailed effects of ZEA treatment on the ovaries and follicles from the early stages of germ cell formation, to postnatal folliculogenesis in newborn and adult females and on the GCs.

**FIGURE 2:**
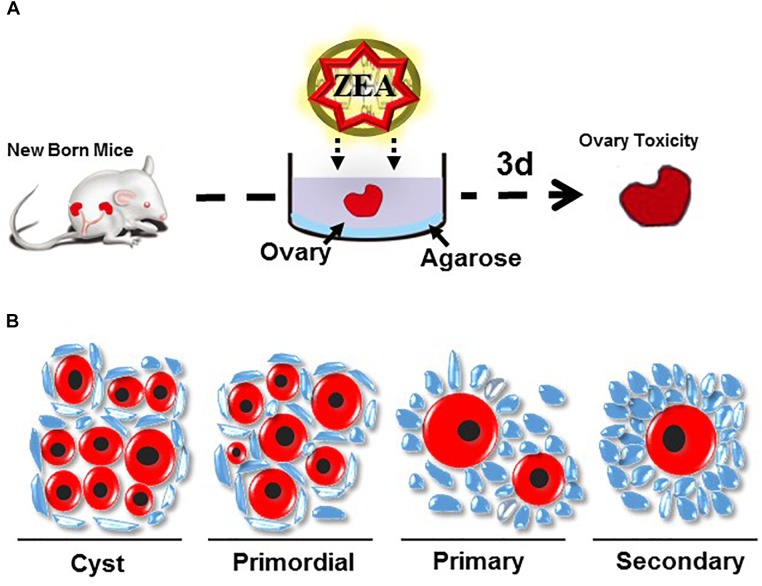
**(A)** Method of ovary culture *in vitro* **(B)** Follicle morphology in mouse ovary.

The mammalian ovary is involved in the development, maturation, and release of matured oocytes for fertilization and in the synthesis and secretion of hormones that are necessary for follicular development, menstrual/sexually desired cyclicity, and the maintenance of the reproductive system (Araujo et al., 2014). Follicle formation is a highly ordered biological process that occurs from fetal life to early after birth. Establishing the pool of primordial follicles (PFs) is essential for female fertility and any abnormality in the ovarian processes could lead to infertility (Kezele et al., 2002). The formation of the PF pool occurs during the fetal stage and shortly after birth (De Felici et al., 2005). Loss of germ cells might occur during each of the processes but mainly during cyst breakdown after birth (De Felici et al., 2005). Hence, exposure to 10 or 30 μM ZEA impairs the establishment of the PF population (Figure 2) and significantly decreases the size of the ovarian PF pool (Zhang et al., 2017a). Exposure of developmental gonads to ZEA *in vitro* could affect the entry and progression of mammal oocytes to meiotic prophase I and promote the cyst breakdown of germ cells. Early postnatal and perinatal ovarian ZEA treatment could also impair the assembly and promote the activation of PFs (Zhang et al., 2017a). In mice, the oocyte cyst breaks down and the oocytes have a single layer with some flattened pre-GCs during the first few days after birth and eventually form PFs. Defects during the ovary cyst breakdown lead to the formation of impaired PF assembly and/or multiple oocyte follicles (Iguchi et al., 1991; Pepling, 2012; Xu and Gridley, 2013). This process is negatively regulated by progesterone and estrogen. By contrast, the Notch signaling pathway positively regulates the interaction between PGCs and oocytes during PF assembly (Trombly et al., 2009; Vanorny et al., 2014).

Zhang et al. (2012) reported that oocyte cyst breakdown and PF assembly are crucially reduced by treatment with ZEA in newborn murine ovaries *in vitro* (Zhang et al., 2012). ZEA increases the number of TUNEL-positive (a marker of apoptosis) oocytes and the level of transcripts, such as the proapoptotic gene Bax in cells. The effects are associated with the reduced expression of oocyte-specific genes, such as Lhx8, Figla, Sohlh2, and Nobox. Researchers have investigated exposure of the ovaries of newborn mice to ZEA which blocked the DNA demethylation of the 3′ CpG sites of the Lhx8 gene in oocytes. This process is significantly associated with folliculogenesis (Zhang et al., 2012). Folliculogenesis was severely impaired in ZEA-treated ovaries transplanted into immunodeficient mouse kidney capsules *in vitro* (Zhang et al., 2014). Finally, the ER and PR mRNA levels decreased after neonatal ovaries were treated with ZEA *in vitro* (Mu et al., 2015), partly through the PPAR-dependent signaling pathways (Kawano et al., 2014). *In vivo* studies confirmed the *in vitro* results. Newborn female mice that received ZEA orally, showed altered oocyte development and folliculogenesis at later ages (Liu et al., 2017). In particular, ZEA exposure significantly decreased the amount of PFs, in mammals at the puberty or adult stages, possibly by accelerating the dynamic rates of follicle recruitment. This reduced the levels of, or delayed the methylation of the imprinted genes in early ovarian oocytes, and increased the occurrence rate of metaphase II spindle abnormalities in mature oocytes (Zhang et al., 2013a,b; Liu et al., 2017).

The activation of PFs for growth starts immediately upon formation, proceeds with oocyte growth and leads to the proliferation of GCs. The PI3K/PTEN signaling pathway is essential for the activation of ovarian PFs. Other factors such as the CDK inhibitor p27Kip1 and FOXO3a, are crucially involved in such processes (McLaughlin and McIver, 2009; Reddy et al., 2010; Sanchez and Smitz, 2012). The PI3K signaling pathway, functions in the oocytes and GCs of primordial and primary follicles. The activation of the PI3K signaling pathway could activate its component protein AKT, which is a serine and threonine protein kinase that generally enhances cell survival and follicle proliferation; at the same time, PTEN is a negative regulator of the PI3K pathway. Hannon et al. (2015) reported that MEHP treatment promoted early folliculogenesis in cultured murine ovaries (Hannon et al., 2015), most probably through the over activation of the PI3K signaling pathway (Hannon et al., 2014, 2016). Zhang et al. (2014) and Moyer and Hixon (2012) revealed reproductive defects in F1 and F2 adult females previously exposed in utero to high doses of MEHP; such defects include accelerated ovarian senescence due to rapid PF activation (Moyer and Hixon, 2012; Zhang et al., 2014). Interestingly, ZEA treatment induced the expression of ovarian cancer-related genes through the PTEN/PI3K/AKT signaling pathway in *E. asinus* GCs *in vitro* (Zhang et al., 2018a).

### Effects of ZEA on Ovarian Secretion

The ovaries are mammalian reproductive organs that are composed of follicles of various sizes. Early stages of ovarian follicular growth mainly depend on the development of follicular GCs and oocytes, which communicate and support one another. Steroids secreted by GCs are essential to the function and normal development of many organs. At present, limited information is available with regard to the influence of ZEA exposure on mammalian ovarian folliculogenesis in experimental animals and livestock (Cortinovis et al., 2013). Porcine and *E. asinus* are more sensitive to ZEA exposure than mice (Kuiper-Goodman et al., 1987; Minervini and Dell′Aquila, 2008). Substantial species differences in biotoxicity exist in animals (Nebbia, 2001). Systematic studies on ZEA are lacking and the action modes of ZEA remain unclear. Researchers used a transcriptome analysis to assess the species-specific toxicity of ZEA; in brief, the ovarian GCs of sow, *E. asinus*, and mice were treated with 10 and 30 μM ZEA *in vitro* and cultured for 72 h (Zhang et al., 2018a,b). Exposure to 10 μM ZEA remarkably altered the expression of the tumorigenesis-associated genes in *E. asinus* GCs (Zhang et al., 2018a), mitosis-associated genes in porcine GCs, and steroidogenesis-associated genes in murine GCs (Zhang et al., 2018b). Interestingly, the number of differentially expressed genes in *E. asinus* GCs treated with 10 μM ZEA was 7254, which is higher than the number of related genes (480) in porcine GCs exposed to the same ZEA dose (Zhang et al., 2018a,b). Hence, ZEA may have a stronger toxic effect on *E. asinus* than on swine (Zhang et al., 2017b, 2018a). The mRNA expression of cancer-promoting genes in *E. asinus* and murine GCs exposed to 30 μM ZEA, was remarkably upregulated (Zhang et al., 2018a). Notably, ZEA treatment remarkably decreased the expression of P53 genes in *E. asinus* GCs. Moreover, exposure to 30 μM ZEA significantly changed the mRNA expression of inflammatory-related genes in porcine GCs. These results illustrate the effect of ZEA dosage on mRNA levels among porcine, *E. asinus*, and murine ovarian GCs *in vitro*.

## Discussion

Zearalenone, a major *Fusarium* mycotoxin, exerts systemic adverse effects on most mammalian species. *In vivo*, *Fusarium* mycotoxins can lead to a follicular growth disorder, ovulation, atresia, and the onset of puberty. The metabolism of mycotoxins, produced by animals, also exert adverse effects. *In vitro*, mycotoxins have direct adverse effects on ovarian primordial follicles by inhibiting the proliferation of germ cells and altering the maturation of oocytes and other functions, such as steroid production of gonadotropin. Considering that ZEA is toxic and ubiquitous, it only poses potential danger to domestic mammals when absorbed in high doses or through prolonged exposure (Diekman and Green, 1992; Obremski et al., 2003).

Zearalenone exposure affects the reproductive function and structural parameters. Swine are the most sensitive domestic species, followed by ruminants, while birds are the most resistant species (Kuiper-Goodman et al., 1987; Olsen et al., 1987). Of all the maturity stages, the pre-pubertal stage in porcine is the most sensitive to ZEA treatment (Bohm, 1992). The adverse changes induced by ZEA exposure depend on the time of administration, which is related to the estrous cycle of gilts and the administered dose (Bohm, 1992). Vulvovaginitis syndrome has been reported in young swine and mature sow (Olsen and Kiessling, 1983). In extreme cases, vaginal and rectal prolapses could occur. Several atresic follicles are present in swine ovaries, and oocyte degeneration occurs (Obremski et al., 2003). However, there are few reports on the clinical symptoms of ZEA exposure in *E. asinus*. Limited data are available regarding the metabolism of ZEA in *E. asinus* (Minervini et al., 2006). The effect of feed contaminants on hypo-fertility cases can therefore not be excluded.

Zearalenone cytotoxicity seriously affects the reproduction (Kiang et al., 1978; Mehmood et al., 2000; Nikov et al., 2000), immunity (Abbes et al., 2006a,b; Luongo et al., 2006), endocrine activities (Mueller et al., 2004), and inheritance (Kouadio et al., 2005) of animals. ZEA treatment impairs the development and steroidogenesis of ovarian GCs (Zhang et al., 2017b), thereby indirectly affecting mammalian fertility (Zhang et al., 2017a). However, most studies on the effect of ZEA toxicity on female reproduction have mainly focused on a single species (Tiemann et al., 2003; Minervini et al., 2006; Ranzenigo et al., 2008; Zhu et al., 2012). Few systematic studies have been conducted among species. Zhang et al. (2018a,b) differentiates the effect of ZEA on GCs among porcine, *E. asinus*, and mice. Furthermore, porcine GCs are more sensitive to ZEA than murine GCs (Zhang et al., 2018b). In addition, *E. asinus* GCs may be more sensitive to ZEA than porcine GCs (Zhang et al., 2017b, 2018a); these findings contradict our previous results. These investigations are the first to report that ZEA potentially and directly changes mRNA abundance and leads to acute inflammation *in vitro*. Moreover, ZEA is an endocrine-disrupting chemical that affects the reproductive performance of domestic animals. Thus, ZEA should also be of great interest to further develop studies that focus on the absorption, metabolism, and eventual storage, in order to better understand its bioavailability. More data on the occurrence and contents of ZEA in feed are needed to improve exposure assessment for domestic animals, especially for swine and *E. asinus*. Studies in domestic and laboratory animals should be initiated to promote the establishment of a safe level of ZEA in different compounded feeds and feed materials, particularly for pigs or *E. asinus*, as they are considered to be the most sensitive animal species, followed by dose-effect studies in other (economic) animals.

## Author Contributions

G-LZ, J-LS, and X-SZ designed and wrote the manuscript. Y-LF collected the data.

## Conflict of Interest Statement

The authors declare that the research was conducted in the absence of any commercial or financial relationships that could be construed as a potential conflict of interest.
